# Sacral neuromodulation for female pelvic floor disorders

**DOI:** 10.1080/2090598X.2019.1589930

**Published:** 2019-04-18

**Authors:** Ahmed S. El-Azab, Steven W. Siegel

**Affiliations:** a Section of Female Urology and NeuroUrology, Assiut University Urology Hospital, Assiut, Egypt; b Minnesota Urology Centers for Continence Care and Female Urology, Woodbury, MN, USA

**Keywords:** Sacral neuromodulation, neuromodulation, female pelvic floor disorders, overactive bladder, urinary retention, pelvic pain

## Abstract

**Objective**: To systematically review available studies on the effectiveness and safety of sacral neuromodulation (SNM) in women with various pelvic floor disorders not responding to more conservative treatment, as SNM is indicated in such women.

**Methods**: Data source: We did a systematic review through the PubMed and the Cochrane Library according to the Preferred Reporting Items for Systematic Reviews and Meta-Analyses (PRISMA) statement from 1998 to 2018 in English using the keywords ‘Sacral Neuromodulation’ and ‘Sacral Nerve Stimulation’.

**Study selection**: Randomised controlled trials and prospective studies were selected, with a minimum sample size of 20 patients and ≥6 months of follow-up.

**Results**: We identified 19 articles. A ≥50% reduction in symptoms qualifies the patient for a permanent implant. Several advances have been introduced into SNM to decrease the invasiveness of the procedure, including a smaller implantable pulse generator battery (improved comfort) and better localisation of the lead wire (improved outcome). The literature reports success for overactive bladder (OAB) to range between 56% and 68% (up to 80%). We report a 5-year therapeutic success rate of 67%. In our previous studies, 38% of our patients with urge urinary incontinence achieved complete continence at 60-months follow-up, with a therapeutic response rate of 57%. Effectiveness in patients with urinary retention and faecal incontinence are about 70% and 85%, respectively. Effectiveness in interstitial cystitis/bladder pain syndrome appears to be lower compared with OAB.

**Conclusion**: SNM is a safe and effective therapy for women with various pelvic floor disorders.

**Abbreviations:** BONT: botulinum toxin; FDA: USA Food and Drug Administration; FS: Fowler’s syndrome; FI: faecal incontinence; IC/BPS: interstitial cystitis/bladder pain syndrome; ICIQ-OABqol: International Consultation on Incontinence Modular Questionnaire-Overactive Bladder Symptoms Quality of Life; INS: implantable neurostimulator; OAB: overactive bladder; PET: positron emission tomography; PNE: peripheral nerve evaluation; PRISMA: Preferred Reporting Items for Systematic Reviews and Meta-Analyses; PTNM: posterior tibial nerve modulation; PVR: post-void residual urine; QoL: quality of life; RCT: randomised controlled trial; SNM: sacral neuromodulation; (U)UI: (urgency) urinary incontinence

## Introduction

The pelvic floor is a complex of muscles, ligaments and fascia that form a multi-layered structure in the inferior pelvis. The pelvic floor has several important tasks: as part of the ‘core’ group of muscles for locomotion and balance, pelvic visceral structural support and control, and sexual functioning. Pelvic floor dysfunction in women may manifest in diverse clinical syndromes including urinary urgency frequency syndrome, urgency urinary incontinence (UUI), forms of urinary retention, bowel dysfunction, pelvic pain, and sexual dysfunction [1].

Pharmacotherapy and behavioural therapy are the standard first-lines of treatment for most pelvic floor dysfunctions. However, these may be suboptimal because of anticholinergic side-effects, insufficient therapeutic benefit, and poor long-term patient compliance. Therapeutic options for patients with refractory overactive bladder (OAB) or those who cannot tolerate pharmacotherapy are limited. Although augmentation enterocystoplasty can be very successful in alleviating symptoms for some patients, the operation is associated with significant short- and long-term complications including: bowel obstruction, fistula, malabsorption, rupture, mucous retention, recurrent infections, and incomplete bladder evacuation requiring clean intermittent catheterisation (CIC) [2].

Sacral neuromodulation (SNM) uses electrical modulation to affect the physiological response of the bladder and other pelvic viscera. In 1997, the InterStim device (Medtronic, Minneapolis, MN, USA) was approved by the USA Food and Drug Administration (FDA) to treat UUI. Later it was approved for urinary urgency frequency syndrome and non-obstructive urinary retention, and finally in 2011 for faecal incontinence (FI). However, SNM has not been FDA approved for treatment of chronic pelvic pain.

Changes in SNM devices, techniques, and technology have made the procedure minimally invasive and able to be performed in an outpatient setting. SNM is becoming more cost-effective thanks to the use of better lead placement techniques; programming, including cycling; and the near future introduction of rechargeable batteries; body compliant leads; and MRI compatibility; all potentially contributing to longer battery life and fewer re-operations, which is reflected in patient satisfaction and improvement of the overall benefit of the device [3].

In recent years several studies have assessed the effectiveness of SNM in the treatment of various female pelvic floor disorders. These studies have reported a great discrepancy in terms of definition of outcome, assessing symptom severity and bother, and definition of cure. Most of the previous studies did not standardise the severity of incontinence or the definition of cure or improvement. Comprehensive evaluation of both subjective and objective outcomes and assessment of patient satisfaction has not been conducted systematically. To understand the current evidence for the use of SNM for these indications, we reviewed outcomes for FDA-approved indications (OAB and non-obstructive urinary retention, FI), as well as other ‘off-label’ uses such as pelvic pain, constipation, and neurogenic bladder.

## Methods

**Figure 1.**
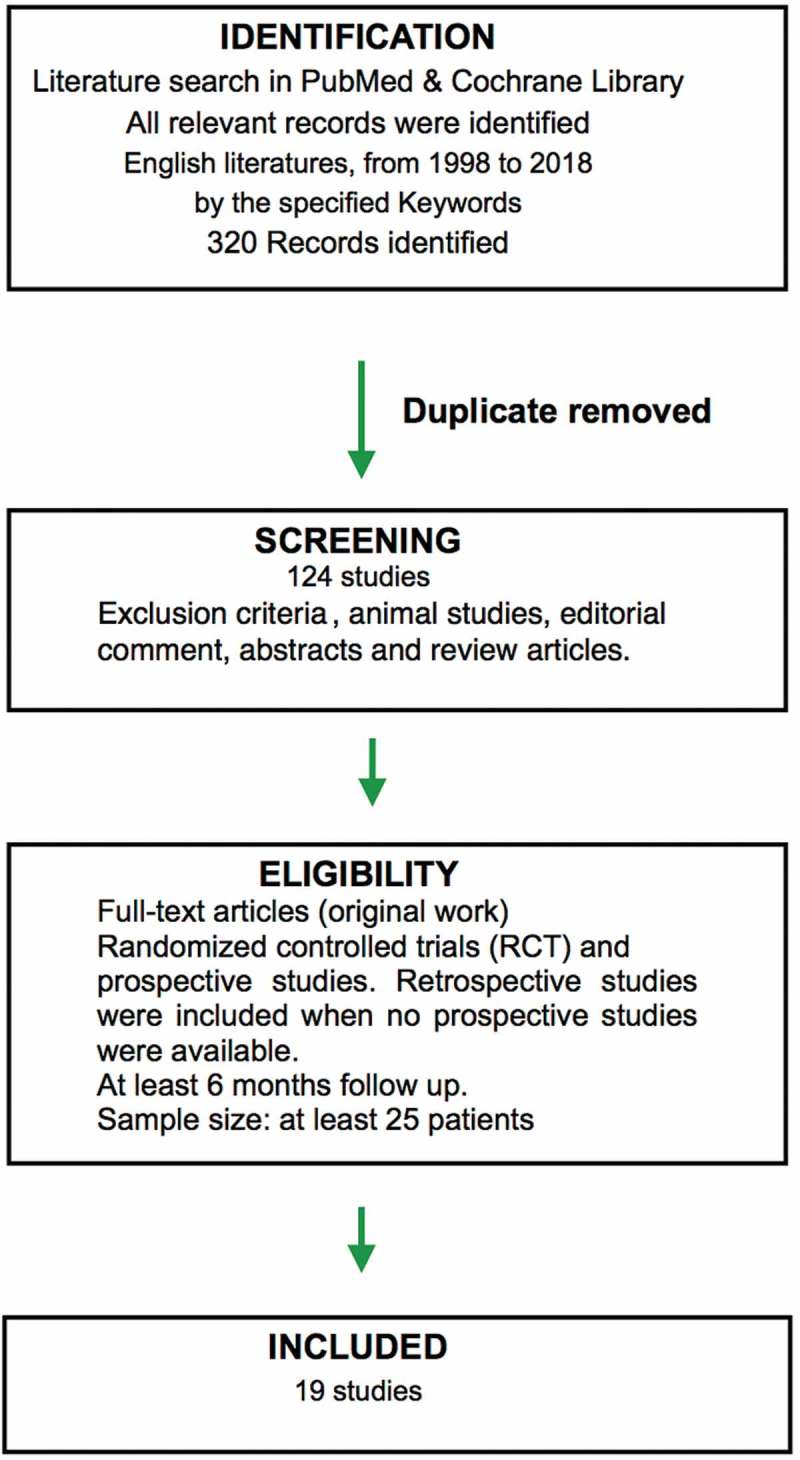
PRISMA flow diagram of the selection of studies.

We conducted a literature search using PubMed and Cochrane Library according to the Preferred Reporting Items for Systematic Reviews and Meta-Analyses (PRISMA) statement [4]. The literature search was from 1998 to 2018, in the English language and using the keywords: ‘sacral neuromodulation’ and ‘sacral nerve stimulation’, ‘female pelvic floor disorders’, ‘lower urinary tract symptoms’, ‘overactive bladder’, ‘urinary retention’, ‘chronic pelvic pain’, and ‘painful bladder syndrome’. We limited our search to randomised controlled trials (RCTs) and prospective studies. Retrospective studies were included when no prospective studies were available. We limited our search to studies assessing the effectiveness and safety of SNM in various female pelvic floor disorders with a good sample size and acceptable follow-up period. After applying these criteria, a total of 124 papers were eligible to be included. The authors then evaluated the articles based on study design, sample size (≥25 patients), and outcome measures. Studies with heterogeneous patient populations or those with no preoperative stratification of patients into groups based on the specific indications were excluded. Finally, 19 articles were included in our systematic review (Figure 1).

## Results

### Indications, patient selection and effectiveness of SNM

#### OAB

Typically, clinicians initially offer behavioural therapies for the treatment of OAB, but if these strategies prove insufficient, patients are started on oral pharmacotherapy. If both are inadequate or cause unacceptable side-effects, clinicians may refer to the AUA Guidelines that suggest ‘third-line’ treatments: botulinum toxin (BONT), SNM, or posterior tibial nerve modulation (PTNM) [5]. As a rule, conservative options should be exhausted by giving an adequate trial before consideration of a third-line treatment. Discontinuation of anticholinergic medications due to adverse effects such as dry mouth, constipation or confusion is common.

In a large part, the decision to select one therapy over others at this stage depends on physician and patient preferences. More evidence from clinical controlled trials is needed to objectively distinguish between multiple options for a particular set of symptoms. The AUA Guidelines have included both SNM and PTNM (Grade C recommendation) and intradetrusor onabotulinumtoxinA (100 U) (Grade B recommendation) as third-line treatments for patients with non-neurogenic OAB, refractory to behavioural therapy and pharmacotherapy. These recommendations were based on RCTs of SNM for urgency/frequency and UUI demonstrating safety and effectiveness along with subsequent follow-up studies showing a durable response.

Several randomised trials have shown short-term improvement in OAB symptoms with PTNM, although there is scant data on long-term effectiveness. PTNM can be offered to any patient with OAB who has failed medical treatment. There are few contraindications to the use of PTNM: those with a cardiac pacemaker and pregnancy. It is more effective for those with mild-to-moderate symptoms. PTNM requires 3 months to determine success.

Potential BONT patients must be aware and prepared to deal with the most common complications of recurrent UTI and urinary retention requiring a period of CIC. PTNM can be instituted for elderly and frail patients who are poorer candidates for SNM or BONT.

We included in our present review a total of 10 reports assessing the effectiveness of SNM on OAB symptoms in women with refractory OAB (Table 1 [6–15]), five RCTs, three prospective studies, and two retrospective studies [6–15]. In addition, Tutolo et al. [16] recently published a systematic review in the *European Journal of Urology* about the safety and effectiveness of the two types of neuromodulation therapy for refractory OAB: SNM and PTNM. They reported that a ≥50% improvement in leakage episodes ranged between 29% and 75%. Overall, the dry rate (zero pads) ranged between 47% and 56%.

**Table 1.** Description of included studies for efficacy of SNM in patients with refractory OAB patients.

Reference	Design	Duration of follow-up, months	Cure rate
Schmidt et al., 1999 [8]	RCT, 34 patients (test stimulation results determined eligibility for randomisation into a stimulation [treatment] or delay [control] group.)	6	No. of leakage/24 h: mean (SD) 2.6 (5.1) vs 11.3 (5.9)
Weil et al., 2000 [13]	RCT (multicentre), 43 patients	Median 18	Mean leakage episodes improved by 88% Leakage severity improved by 24% Pad usage improved by 90%
Hassouna et al., 2000 [7]	RCT (multicentre), 51 patients	6	No. of voids/day improved by: 56% vs 4% Voided volume/void improved by: mean (SD) 226 (124) vs 123 (75) mL Degree of urgency improved by: mean (SD) 1.6 (0.9) vs 2.3 (0.5) Bladder volume improved by: mean (SD) 227 (104) vs 325 (185) mL
Amundsen et al., 2016 [6] (ROSETTA trial)	Multicentre RCT to compare SNM (*n* = 192) and Onabotulinum A (*n* = 194)	6	Significant improvement in daily episodes of UUI: – 3.25 (–3.64/–2.87) for SNM vs – 3.89 (–4.26/–3.52) for BONT; *P* = 0.01
Siegel et al., 2015 [10]	RCT, multicentre trial, 147 patients SNM (*n* = 70) vs standard medical therapy (*n* = 77)	6	Success rate significantly higher (61%) in the SNM group than in the standard medical therapy group (42%); *P* = 0.02
Siegel et al., (2016) [11]	Prospective (multicentre), 272 patients	36	Complete continence achieved in 43% of cases
Siegel et al., 2018 [9]	Multicentre prospective study on 340 patients (272 had permanent implant)	60	Success rate = 67%. UUI episodes a mean (SD) reduction from baseline of 2.0 (2.2) leaks/day and patients with urgency-frequency had a mean (SD) reduction of 5.4 (4.3) voids/day (*P* < 0.001). There was improvement in all ICIQ-OABqol measures (*P* < 0.001)
van Kerrebroeck et al., 2007 [15]	Prospective multicentre, 152 patients	60	Mean leaking episodes/day decreased from a mean (SD) of 9.6 (6.0) to 3.9 (4.0) at 60 months. For patients with urgency frequency the mean voids/day decreased from a mean (SD) of 19.3 (7.0) to 14.8 (7.6)
Sutherland et al., 2007 [12]	Retrospective (single centre), 83 patients	Mean 22	Mean decreases: 4.3 voids/24 h; 1.0 void/night; 4.4 leaks/24 h; and 2.3 pads/24 h (all *P* < 0.05)
Peeters et al., 2014 [14]	Single centre retrospective study, 217 patients	Mean 46	Success and cure rates: 70% and 20% for UUI, 68% and 33% for urgency frequency syndrome 73% and 58% for idiopathic retention

Comparing SNM to BONT was the focus of the Refractory Overactive Bladder: Sacral Neuromodulation vs Botulinum Toxin Assessment (ROSETTA) trial for the treatment of women with refractory UUI [6]. The study concluded that: ‘Among women with refractory UUI, treatment with onabotulinumtoxin A compared with SNM resulted in a small daily improvement in episodes that although statistically significant is of uncertain clinical importance. In addition, it resulted in a higher risk of urinary tract infection and need for transient self-catheterizations’. The ROSETTA trial was an open-label randomised study conducted at nine sites. Eligible patients had to have had at least six episodes of UUI on a baseline 3-day voiding diary. The study excluded patients with frequency and urgency without UUI. Patients were randomised to receive intradetrusor injection of 200 U BONT (192 patients) on an outpatient basis or SNM (189 patients). The first stage of SNM lead placement was done in the operating room. During a 7–14 day test phase, study subjects with a ≥50% reduction in the number of episodes of UUI in a 3-day bladder diary went on to neurostimulator implantation. There was greater improvement in daily UI episodes (3.9 vs 3.3, *P* = 0.01), as well as in symptomatic bother scores (46.71 vs 38.5, *P* = 0.002 on the OAB questionnaire short-form) for the BONT arm. Complete symptom resolution favoured BONT (20% vs 4%, *P* < 0.001). A >75% improvement occurred in 50% of the BONT group vs 27% of the SNM group. There was no statistically significant difference in quality of life (QoL). Six patients in the SNM group (3%) had their device revised or removed during the 6-month period. In the BONT group, 8% required CIC at 1 month, 4% at 3 months, and 2% at 6 months. The threshold for CIC was a post-void residual urine volume (PVR) of >300 mL or >200 mL with symptoms of incomplete voiding. By 6 months the risk of UTI was greater in the BONT group (35%) than in the SNM group (11%). Criticism of this study design include the use of a non-clinically relevant dose of BONT for idiopathic OAB (200 vs 100 U), exclusion of OAB-dry patients thus allowing no conclusion relative to that group, and routine use of an SNM lead (model 3093), which has been discontinued by the manufacturer due to its inferiority to the more commonly used alternative (model 3889) lead [17].

Siegel et al. [11] reported the findings at 6 months of follow-up of patients randomised to SNM (70 were randomised to SNM using the optimised staged technique) or to standard anti-muscarinic medications (77 patients). At 6 months, the OAB success rate was 61% in the SNM group compared to 42% in the standard medical therapy group. In addition, <8 voids/day was achieved by 61% of SNM patients compared to 37% of the standard medical therapy patients. In a recently published multicentre study, Siegel et al. [9] evaluated the success and safety of SNM at 60 months after InterStim in 340 patients with OAB, with 272 proceeding to permanent lead implant. Success was defined as a ≥50% improvement in average leaks or voids/day, or return to normal voiding, defined as <8 voids/day. They reported a 60-month therapeutic success rate of 67%. Amongst patients with UUI, complete continence at 60 months follow-up was achieved in 38% of them. Amongst patients with urgency–frequency, a therapeutic response rate of 57% was observed. Those patients had an average reduction of 4.4 voids/day from baseline (*P* < 0.001). The patients had a significant improvement in QoL measurements (International Consultation on Incontinence Modular Questionnaire [ICIQ]-Overactive Bladder Symptoms Quality of Life [OABqol]). The most common device-related adverse events were an undesirable change in modulation parameters in 22%, implant site pain in 15%, and loss of effectiveness over time in 36 (13%).

We believe that SNM is the superior third-line therapy for several important reasons. SNM is more holistic, as it restores function to the patient instead of creating an equal and opposite dysfunction to balance an existing one, as does BONT. It can work well for both OAB-wet and -dry, whilst BONT is indicated only for UI. The potential therapeutic benefit of SNM is not limited to the bladder. It can help manage associated bowel dysfunction when co-existing with genitourinary complaints. SNM does not cause urinary retention or UTI as can BONT. BONT can be used immediately after if SNM fails but not vice versa, as the effects of BONT must be reversed before SNM is attempted, to minimise false negatives. Finally, SNM is known to be an effective, safe and completely reversible therapy that can last for decades.

#### Pelvic pain in women

Pain is the hallmark symptom of interstitial cystitis/bladder pain syndrome (IC/BPS). IC/BPS is no longer considered a bladder disorder but is recognised as a complex chronic pain syndrome [18]. Pain is reported in the suprapubic region, urethra, vagina, rectum, and sometimes in extragenital locations such as the lower abdomen and lower back. Patients with IC/BPS commonly void to relieve pain associated with bladder filling (whilst those with OAB void to prevent UI). It is therefore essential to control pain as well as urgency in such patients [19].

SNM has not been approved for IC/BPS. However, studies have reported the effectiveness of SNM in alleviating pain in such patients. We included in our present review two prospective studies assessing the effectiveness of SNM in women with IC/BPS [20,21]. Comiter [20] performed a prospective single centre study to evaluate SNM in 25 patients with refractory IC. Although the sample size was small and the follow-up was only 14 months, the study reported that pain decreased from 5.8 to 1.6 of a score of 10 (*P* < 0.01) and symptom improvement was sustained in 94% of cases. Whitmore et al. [21] performed a multicentre prospective observational study of 33 patients with intractable IC who received SNM. Analyses of voiding diary data showed statistically significant improvements in frequency, pain, average voided volume and maximum voided volume (*P* < 0.05). These studies are both observational studies with relatively small sample sizes. The AUA Guidelines suggest that patients should start appropriate oral agents and instillation programmes, and optimise the pain control regimen. If they still have uncontrolled voiding dysfunction despite this multimodal approach, SNM can be considered. Patients must be aware that there is no significant evidence to use SNM for pain other than voiding symptoms. It must be clear to patients that SNM for IC/BPS is only indicated for voiding symptoms and that any effect on pain is simply unpredictable.

Some of the negative assumptions related to SNM for pain could be based on conclusions drawn from experience prior to the advent of routine optimisation of lead placement. In the authors’ opinion, precise lead placement may be less important for conditions like FI or OAB-wet, for which SNM has proven to be very robust. Lead placement may in reality be much more demanding for patients with sensory dysfunctions such as IC/PBS, where collateral stimulation of anything other than the relevant portion of the sacral nerve is poorly tolerated [22].

#### Urinary retention in women

Blaivas et al. [23] reported that the incidence of BOO amongst women presenting with LUTS is 8.3%. However, it is widely accepted that the true incidence is underestimated. One of the challenging aspects of urinary retention in women is how it is determined. There is no simple and clear definition of urinary retention in women in the literature. The diagnosis is often based on a combination of symptoms, PVR and pressure-flow studies [24]. Management of urinary retention in women is challenging. Many women are treated with α-blockers, urethral dilatation, CIC or permanent drainage. Women often describe CIC as uncomfortable, and they generally would prefer an alternative line of therapy.

We included in our review three studies assessing the effectiveness of SNM in women with urinary retention [14,15,25]. Dasgupta et al. [25] reviewed the results of SNM in a group of 26 women with Fowler’s syndrome (FS). After 72 months follow-up, they reported an impressive success rate, with 20/26 patients (77%) voiding spontaneously without need for CIC. Dasgupta and Fowler [26] analysed the urodynamic data of those women with refractory urinary retention due to FS who underwent SNM. They found that sphincter activity does not change after SNM, whilst detrusor contractility increases. They postulated that this increase in contractility led to improved voiding in those patients as a result of SNM. FS, which mostly affects young women, may represent the most common identifiable cause of urinary retention in women. Retention is due to primary abnormality of the striated urethral sphincter, with an abnormal increase in sphincter electromyographic activity associated with elevated maximum urethral closure pressure. Prolonged increase in the urethral closure pressure in those women leads to detrusor failure (the detrusor contraction is inhibited) and suppression of bladder sensation. That is why retention in those women is associated with large bladder volumes (>1 L) and is not associated with a severe desire to void as might be expected [26]. It has been postulated from several functional MRI and positron emission tomography (PET) studies that the brain response to bladder afferents is attenuated in patients with FS. A study using PET has documented the attenuation of the brain response to a full bladder in patients with FS. The study documented the deactivation of the regional brain matrix regions implicated in control of the bladder (the periaqueductal grey and thalamus), an action that was reversed by SNM. With SNM afferent activity reaches the periaqueductal grey, presumably because SNM blocks urethral inhibition of afferent information flow from the bladder, thus enabling voiding [27,28].

For urinary retention, van Kerrebroeck et al. [15] reported a success rate of 50% in average catheterisations per day and 71% in average catheterised volume per day, and the difference from baseline was statistically significant in 31 patients with urinary retention. Peeters et al. [14] showed a >50% success in at least 1 voiding diary parameter of 73% and ‘cure rates’ of 62.5% and 53% in FS and non-FS idiopathic retention.

#### Bowel dysfunction

FI is defined as the involuntary loss of stool. It is one of the most psychologically and socially debilitating and humiliating conditions. The exact prevalence of this condition is unknown, but published rates have ranged from 2% to 3% amongst community dwelling subjects. Vaginal delivery is the most common predisposing factor to FI in an otherwise healthy woman. Vaginal delivery may result in mechanical anal sphincter disruption, or may cause damage to the pudendal nerve through overstretching and/or prolonged compression and ischaemia. The overall anal incontinence rate after traumatic vaginal birth ranges between 9% and 45%. Inadequate repairs of obstetric sphincter injuries may contribute to delayed symptoms of FI [29]. Age may also play a role in the development of FI. Other aetiologies for FI include: congenital abnormalities, spinal cord injury, inflammatory bowel disease, anal surgery, medical conditions such as diabetes mellitus, stroke, spinal cord trauma, and degenerative disorders of the nervous system. These conditions may alter normal sensation, feedback, or function of the complex mechanism of anal continence [30]. Non-surgical treatment options for those patients include dietary modification, pharmacological therapy with anti-motility agents, biofeedback, injectable bulking agents, and radiofrequency application to the anal sphincter [31]. The InterStim device is FDA approved for the treatment of FI.

Several randomised trials have reported excellent effectiveness of SNM in patients with FI. We included in our present review two studies assessing the effectiveness of SNM in women with bowel dysfunction. Wexner et al. [32] reported on a prospective multicentre study of 133 patients with FI, and 120 of them underwent permanent implantation. The therapeutic success rate was 85% at 24 months (*P* < 0.001) and 41% of the patients achieved 100% faecal continence.

In addition to treatment of FI, SNM shows good results for refractory constipation. Despite the absence of evidence-based data, available evidence suggests that SNM therapy may provide beneficial results for selected patients with idiopathic slow or normal transit constipation and obstructive defaecation [33].

#### Neuropathic bladder

SNM has not been approved by the FDA for neurogenic bladder disorders. Few studies have shown that SNM is effective in groups of patients with neurogenic bladder disorders. However, most of these studies have major methodological problems, such as small sample sizes and heterogeneous patient populations. An important issue that has not been addressed is the change in the response with time, especially with progressive neurological disorders such as disseminated sclerosis. One would expect less effectiveness over time. As mentioned before, most trials evaluating SNM in neuropathic bladder did not meet our inclusion criteria. Lombardi and Del Popolo [34] described 24 patients with incomplete spinal cord injury with neurogenic LUTS for whom SNM was performed and with a mean follow-up of 61 months. They divided their study patients into two groups: Group 1, the retention group included patients with urinary retention (*n* = 13) and Group 2 included patients with OAB symptoms (*n* = 11). Interestingly they did not describe any urodynamic criteria for their study patients. In the retention group, nine of 13 patients reported a 50% improvement in baseline voiding parameters, with a significant decrease in the number of catheterisations and a significant increase in the frequency of voids and voided volume. At the end of the study, 38% of their patients no longer required catheterisation for bladder emptying. Amongst the patients with OAB, an 80% reduction in daytime frequency was observed, with three of seven patients with previous UUI remaining completely dry during the study period. This study illustrates the dual effectiveness of SNM for the spectrum of voiding dysfunction found in patients with spinal cord injuries. However, the small sample size, especially after patients’ stratification, prevents the generalisability of the data of this study. Other trials of SNM for neurogenic bladder have been less promising.

### The initial trial period

We recommend complete urological evaluation including: medical history, physical examination, bladder diary, urine analysis, and when indicated, urine culture, urinary tract ultrasonography, urethrocystoscopy, and urodynamic evaluation if findings will change potential therapy. The patient should complete a baseline voiding diary. If the SNM is being conducted for FI, details of these episodes along with consistency of stool and number of daily bowel movements should also be documented with a diary. It is important to counsel the patient, so they know what to expect during the procedure and help reduce patient anxiety, as it may be performed without sedation in the case of a peripheral nerve evaluation (PNE).

There are two different types of trials for patients undergoing test modulation with SNM, the PNE and staged lead implantation. The PNE is more simple and basic, and involves placement of a temporary monopolar lead, which can be performed in the office under local anaesthesia. The lead can be easily removed at the end of a 3–10 day trial period. The average onset of action is ~3 days with a maximum of 9 days. Therefore, a test stimulation of a maximum of 2 weeks seems sufficient [35].

A disadvantage is the potential for migration of the leads, thereby decreasing the accuracy of the trial in determining whether a patient will benefit from the therapy and higher false negative results. For the initial trial phase, the author uses both types of test modulation: choosing a PNE or staged trial procedure is based on the patient’s preference and condition. FI may only happen once per week, and in this case a staged procedure would be more likely to capture the benefit when episodes are less frequent. During the staged trial, a quadripolar tined lead is positioned as the first stage with placement of the implantable neurostimulator (INS) as a second stage, if the trial proves to be successful. Here, the lead is placed more accurately, it is less likely to migrate during the trial, and the programming options during the trial phase are more plentiful and robust. However, the staged procedure requires an operating room and anaesthesia. A PNE may be performed using fluoroscopy or anatomical landmarks to determine placement of the temporary leads. The author uses fluoroscopy in PNE to enhance lead placement. We typically conduct the staged lead implantation over a period of 3–4 weeks. A ≥50% improvement of the relevant symptoms qualifies patients for the INS during the second stage. If there is a <50% improvement, the lead is removed at this time.

### Safety of SNM

Complications from SNM are usually minor and reversible without surgery. Recent rates of device-related adverse events and surgical intervention are significantly lower than in previously published studies using older techniques and devices [36]. This is attributable to further refinements of the lead placement technique and better patient selection parameters. Change or loss of effectiveness over time, pain at the INS or lead site, and painful stimulation are amongst the common side-effects of SNM. Risk factors for adverse events included trauma, a change in body mass index, and enrolment in a pain clinic. Pain at the implant site ranged between 15% and 42%, and surgical revision rate between 9% and 33%. The most common reason for surgical revision was pain at the implant site [16].

## Discussion

The technique of SNM has been discussed in detail elsewhere. Several advances have been introduced into SNM to decrease the invasiveness of the procedure with a smaller IPG battery (improved comfort) and better localisation of the lead wire (improved outcome) [37].

All reports on SNM in patients with neurogenic bladder dysfunction (adult and paediatric) included heterogeneous CNS pathologies, which limits the usefulness of these studies. However, SNM seems to be beneficial in adults with neurogenic bladder. The randomised studies on which FDA approval for SNM was obtained, excluded patients with neurological conditions such as spinal cord injury and stroke. To date, it is not clear which subgroup of patients with different aetiologies of neurogenic bladder are most likely to benefit. Randomised trials with homogenous pathologies are needed to answer this question. Currently a prospective randomised double-blinded placebo-controlled multicentre study is being conducted in Switzerland looking at the effect of SNM in adult patients with neurogenic bladder [38].

The InterStim device has undergone many technical developments to enhance lead placement and patient comfort. In 2002, a self-anchoring tined lead significantly decreased the invasiveness of the procedure [39]. In 2006, the new IPG, known as the InterStim II, was introduced onto the market and is now the standard. The InterStim II is smaller than the original and was developed in efforts to decrease patient’s discomfort. However, at one-third the volume, this smaller IPG also reduces battery life by almost 50% [40]. There are two types of stylets used to place the lead wire during the procedure. The original straight stylet made from tungsten and the newly modified curved stylet has a smaller diameter and is made from stainless steel (making it slightly more flexible). The lead wire itself has four electrodes, with ‘0’ and ‘1’ being the two deepest. Curved styles are associated with better lead placement, so that the electrodes are in close proximity to the sacral nerve root. Maximising battery life is important to reduce the re-operation rate. Using the lowest effective amplitude (energy) setting helps maximise battery longevity and is, therefore, of importance. The curved stylet better follows the curvilinear course of the sacral nerve root, and is associated with better placement of the deeper electrodes closer to the nerve root, resulting in lesser energy delivered and longer battery life. The third modification in the technique is electrophysiological studies during the procedure. Intraoperative electromyography during lead placement can further improve patient selection and lead placement [41].

## Conclusion

SNM has changed the management of female pelvic floor disorders. Advances in SNM techniques, along with other third-line therapies, allow excellent alternatives to irreversible destructive/reconstructive procedures such as bladder augmentation and urinary diversion. OAB, the most common female pelvic floor disorder, affects millions of women worldwide. When conservative therapies have failed to provide adequate control of symptoms, SNM should be considered as the preferred next step in the treatment algorithm. Multiple long-term follow-up studies have shown SNM to be an effective and safe therapy.
